# Brain conditions mediate the association between aging and happiness

**DOI:** 10.1038/s41598-022-07748-6

**Published:** 2022-03-11

**Authors:** Keisuke Kokubun, Kiyotaka Nemoto, Yoshinori Yamakawa

**Affiliations:** 1 Open Innovation Institute, Kyoto University, Kyoto, Japan; 2 Smart-Aging Research Center, Tohoku University, Sendai, Japan; 3 Department of Psychiatry, Faculty of Medicine, University of Tsukuba, Tsukuba, Japan; 4 ImPACT Program of Council for Science, Technology and Innovation (Cabinet Office, Government of Japan), Chiyoda, Tokyo, Japan; 5 Institute of Innovative Research, Tokyo Institute of Technology, Meguro, Tokyo, Japan; 6 Office for Academic and Industrial Innovation, Kobe University, Kobe, Japan; 7 Brain Impact, Kyoto, Japan

**Keywords:** Neuroscience, Psychology

## Abstract

As the population ages, the realization of a long and happy life is becoming an increasingly important issue in many societies. Therefore, it is important to clarify how happiness and the brain change with aging. In this study, which was conducted with 417 healthy adults in Japan, the analysis showed that fractional anisotropy (FA) correlated with happiness, especially in the internal capsule, corona radiata, posterior thalamic radiation, cingulum, and superior longitudinal fasciculus. According to previous neuroscience studies, these regions are involved in emotional regulation. In psychological studies, emotional regulation has been associated with improvement in happiness. Therefore, this study is the first to show that FA mediates the relationship between age and subjective happiness in a way that bridges these different fields.

## Introduction

As the population ages, the realization of a society in which people can live happily in old age is becoming an increasingly important issue in many countries. This is because it is generally believed that people become unhappy with age^1^. However, many studies using the Subjective Happiness Scale (SHS)^2^, a typical psychological measure of happiness, have contradicted this idea. An Italian study found that happiness under the age of 45 is higher than happiness over the age of 45^3^. In contrast, some studies have shown that happiness increases with age^3–6^. However, more studies have found that happiness does not differ depending on age^2,7–11^. Similarly, a recent study of 4,977 people in nine locations in Europe, the United States, and Australia found no differences in happiness with age^12^. Studies using well-being scales other than SHS have also shown a U-shaped relationship with age^13–16^. There is an argument^17^ that aging does not reduce happiness because people change their life goals to be more realistic as they grow older. It is also argued that as one gains experience with age, one becomes more aware of the positive aspects of life and averts unhappiness^18^. Similarly, most studies with SHS found that there was no gender difference in happiness^8,11,12,19,20^.

However, in the field of neuroscience, studies have shown that the index of the brain indicated by gray matter (GM) and FA decreases with age^21–27^. Studies have also shown that the brain correlates with happiness. For instance, it has been demonstrated that individuals with high SHS and related scales have greater rostral anterior cingulate cortex^28^, insular cortex^29^, and precuneus^30^ as measured by GM volume. Similarly, whole-brain VBM studies on dispositional optimism^31^ and global life satisfaction^32^ have reported positive correlations with GM volume of the medial temporal lobes, including the parahippocampal gyrus. In addition, two structural MRI studies have found that smaller hippocampal volume is related to lower self-esteem^33,34^. Furthermore, several previous functional imaging studies have found that other brain regions, such as the anterior cingulate gyrus and amygdala, were active during the induction of happy emotions^35–39^.

The differences in the regions that correlate with happiness in each study may be due to small sample sizes, cultural differences, and variations in research methods^28,30,40^, although these views have not been empirically confirmed. However, we think the bigger problem lies, not in these regional inconsistencies, but elsewhere. Specifically, if the brain weakens with age, why does the sense of happiness empirically correlate with the brain and not decline with age? To the best of our knowledge, no study has answered this question. One possibility is that it may be another brain index that correlates more directly with happiness, rather than GM volume. For example, previous studies have shown that FA, especially the limbic-thalamo-cortical pathway, is involved in emotional regulation^41–44^. Therefore, in this study, we aimed to clarify the relationship between age, brain, and happiness with the hypothesis that it is not GM that is directly related to happiness, but the FA that represents the integrity of the white matter involved in emotional regulation while communicating the major brain regions.

## Materials and methods

### Subjects

**Figure 1:**
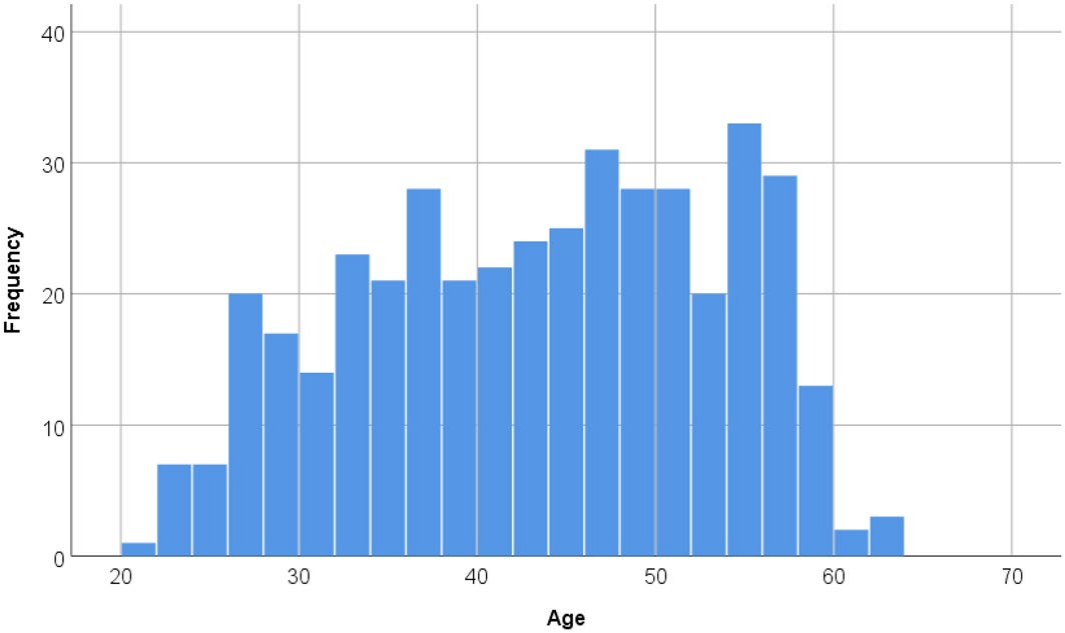
Distribution of age.

From September 2018 to October 2020, a total of 417 healthy participants (107 women, 310 men), 21–63 years of age (mean ± standard deviation (SD) age, 42.9 ± 10.0 years) were recruited in Kyoto and Tokyo. Participants are mainly employed persons gathered by member companies of the BHQ Consortium, which is a study group aimed at maintaining and improving the brain health of individuals. No subjects recruited were likely to have records of neurological, psychiatric, or other medical conditions that could affect the central nervous system. This study was approved by the Ethics Committee of Kyoto University (Approval Number 27-P-13) and Tokyo Institute of Technology (Approval Number A16038) and was conducted following the institutes’ guidelines and regulations. All participants provided written informed consent before participation, and their anonymity was maintained. Figure 1 shows a histogram showing the distribution of age.

### Psychological scales

We use the Subjective Happiness Scale^2^, which was developed to assess subjective happiness and consists of four items assessed on a 7-point Likert scale. For the first two items, respondents were required to rate their general happiness (1 = not a very happy person to 7 = a very happy person) and happiness compared to their peers (1 = less happy to 7 = more happy). The other two items give a brief description of generally happy and unhappy people, and respondents are asked to indicate how much each trait describes them (1 = not at all to 7 = a great deal). The variables used in the analysis were the arithmetic mean values of the four items. The reliability coefficient (Cronbach's α) of the SHS used in this study was 0.834, which is sufficiently high. Recent studies using SHS have shown that people with higher resilience, hope, and positive attitude tend to maintain lower fear and higher happiness during the COVID-19 pandemic^45,46^. The results of these studies indicate that happiness is associated with certain stress tolerances.

The reason why the current study uses SHS is that this scale is one of the most widely used well-being measures internationally. SHS has also been used in recent studies of brain structure in Japan^28,30,47^. However, there is another merit of using SHS. A study by Sato and colleagues^30^ showed that SHS correlates with gray matter volume in the right precuneus, while the same region also correlates with positive and negative emotional intensity and purpose in life. This suggests that if some correlations with SHS can be confirmed in the brain structure, a correlation with other similar well-being indicators can be inferred to some extent. Moreover, some studies based on resting-state functional magnetic resonance imaging found that functional connectivity between the right precuneus and the right amygdala^48^ or between the dorsolateral prefrontal cortex and some networks (decreased primary sensory cortices, decreased default mode network, and increased frontoparietal attention networks)^49^ were positively associated with SHS scores. These indicate that SHS is involved not only in brain structure but also in function.

In addition, especially in studies other than Japan, measures other than SHS are used in studies of brain structure^29,32,50,51^ and function^52,53^. However, not all typical well-being scales are commonly used in brain research. For example, few studies have used the Cantril Ladder Scale^54^, one of the leading measures of overall well-being, to test the correlation of well-being with brain structure and function. Exceptionally, Buhrmann and colleagues analyzed the relationship between the Ladder Scale and cerebellar GM volume but found no statistically significant correlation between the two^55^. One possible reason is that the Ladder Scale has been shown to correlate more strongly with physical affluence than other well-being scales^56–58^. This is because Ladder Scale primes financial security and material possessions as well as nonmaterial factors, and therefore responses to it are affected by material wealth as well as other factors^57^. Based on the above, considering that this study is aimed at Japanese people, SHS, which is a scale for measuring pure happiness and has been used in the same country, is used instead of comprehensive well-being scales.

### Demographic scales

As demographic scales, the body mass index (BMI in kg/m^2^), knowledge work, and annual income were adopted. BMI was computed using height and weight. Knowledge work is a binary variable that takes 1 when “administrative” or “professional/technical” is selected and 0 when either is not selected from the following 13 choices for occupation: “administrative”, “professional/technical”, “office work”, “sales”, “service”, “security”, “agriculture, forestry, and fishery”, "production process”, “transportation/mechanical operation”, “construction/mining”, “carrying/cleaning/packaging”, “student”, and “unemployed”. The annual income was created by allocating 1 to 16 points for each of the following 16 options: "No income", "less than 500,000 yen", "500,000 to 990,000 yen", "1 to 1.49 million yen", "1.5 to 1.99 million yen", "2 to 2.49 million yen". "2.5 to 2.99 million yen", "3 to 3.99 million yen", "4 to 4.99 million yen", "5 to 5.99 million yen", "6 to 6.99 million yen", "7 to 7.99 million yen", "8 to 8.99 million yen", "9 to 9.99 million yen", "10 to 14.99 million yen", and "15 million yen or more".

BMI is used because a negative correlation with GM has been consistently seen in the previous studies^21–24,26^. Therefore, to accurately measure the correlation between GM and age, it is necessary to control the effect of BMI. In studies with SHS as the dependent variable, BMI is also used as a control variable along with sex and age^43,44,64^. Such manner is justified considering the correlation of SHS with health satisfaction^3^ and an evaluation that BMI is the measure as a holistic appraisal of health^65^. On the other hand, the reason why knowledge work is used as a variable is that it is necessary to control the intellect of the respondents because this study deals with the brain. Previous studies investigating the relationship between SHS and brain structure also used variables related to intelligence as covariates^30,63^. In a study by Sato and colleagues who analyzed the relationship between GM volume and SHS, full-scale intelligence quotient (IQ) was used as a covariate in addition to sex and age^30^. On the other hand, in a study by Maeda and colleagues who investigated the relationship between FA and well-being as in this study, a widely used measure of general intelligence, which is different from IQ, was used as a covariate in addition to brain volume^63^. In this study, we decided to use knowledge work instead of IQ as a variable that reflects the more social background of the respondents, which is not sufficiently captured by IQ, although previous studies have shown that knowledge work generally has a high IQ^64^. Income, on the other hand, is used because several studies have shown that SHS and well-being correlate with social status such as income^65,66^. There are also examples of its use in studies of brain structure^67,68^. However, income has not been used in a series of studies investigating the relationship between the brain and well-being discussed above. Therefore, as a trial, in this study, we examine a model in which annual income is added to covariates in addition to BMI and knowledge work. In other words, by controlling BMI, knowledge work, and income, it is thought that the effect of health, intelligence, and social status differences on SHS can be minimized and the correlation of age and brain information with SHS can be estimated more accurately. The adoption of these three variables is consistent in the light of previous studies of brain structure studies that put intelligence into covariates, and with the argument that the main determinants of well-being are mental and physical health, social relations such as occupation, and living standards such as income^69^. However, unlike intelligence, income is not often used in the research of the relationship between happiness and the brain. Also, unlike BMI, which is calculated from height and weight, income information obtained from written answers together with SHS items in the same questionnaire cannot eliminate the effects of common method bias. In addition, the problem of multicollinearity due to a high correlation with knowledge work can occur. Therefore, in this paper, we describe two results with and without income added to the control. However, other demographic variables are not used in this study as they have not been used in previous studies on the relationship between the brain and well-being. For example, many studies have shown the relationship between marital status and well-being^65,70^. Marital status has also often been used in some brain studies^71^. However, it has not been used in the study of the relationship between happiness and the brain shown above. Therefore, there may be discoveries by using the marriage status as a covariate. However, to facilitate the interpretation of the results and comparison with previous studies, these demographic variables will be used at another time rather than in this study which focuses on happiness and the brain structure related to the function of emotional regulation.

### MRI data acquisition

All magnetic resonance imaging (MRI) data were obtained using a 3-T Siemens scanner (Verio, Siemens Medical Solutions, Erlangen, Germany or MAGNETOM Prisma, Siemens, Munich, Germany) with a 32-channel head array coil. A high-resolution structural image was acquired using a three-dimensional (3D) T1-weighted magnetization-prepared, rapid-acquisition gradient echo pulse sequence. The parameters were as follows: repetition time (TR), 1900 ms; echo time (TE), 2.52 ms; inversion time (TI), 900 ms; flip angle, 9°; matrix size, 256 × 256; field of view (FOV), 256 mm; and slice thickness, 1 mm. DTI data were collected by spin-echo echo-planar imaging (SE-EPI) using generalized auto-calibrating partially parallel acquisitions (GRAPPA). The image slices were parallel to the orbitomeatal (OM) line. The parameters were as follows: TR = 14,100 ms; TE = 81 ms; flip angle = 90°; matrix size = 114 × 114; FOV = 224 mm; slice thickness = 2 mm. The baseline image (b = 0 s/mm^2^) and 30 different diffusion directions were obtained with a b-value of 1000 s / mm^2^.

### GM-BMQ and FA-BHQ

T1-weighted images were pre-processed and analyzed using Statistical Parametric Mapping 12 (SPM12; Wellcome Trust Center for Neuroimaging, London, United Kingdom) running on MATLAB R2015b (Mathworks Inc., Sherborn, MA, United States). Each MPRAGE image was divided into gray matter (GM), white matter (WM), and cerebrospinal fluid (CSF) images. The segmented GM images were spatially normalized using a diffeomorphic anatomical registration through an exponentiated lie algebra (DARTEL) algorithm^72^. A modulation step was also incorporated into the pre-processing model to reflect the regional volume and preserve the total GM volume before the warp. As a final preprocessing step, all normalized, segmented, and modulated images were smoothed with an 8 mm full width at half-maximum (FWHM) Gaussian kernel. Intracranial volume (ICV) was calculated by summing the GM, WM, and CSF images for each subject. Proportional GM images were generated by dividing the smoothed GM image by ICV to control for differences in whole-brain volume between participants. These proportional GM images were used to generate the mean and standard deviation (SD) images of all participants. Next, we calculated the GM brain healthcare quotient (BHQ), which is similar to the intelligence quotient (IQ). The mean was defined as BHQ 100, and SD was defined as 15 BHQ points. By this definition, about 68% of the population is between BHQ 85 and BHQ 115, and 95% of the population is between BHQ 70 and BHQ 130. Individual GM quotient images were calculated using the following formula: 100 + 15 × (individual proportional GM–mean)/SD. Next, automatic anatomical labeling (AAL) atlas^73^ was used to extract regional GM quotients and averages across regions to create participant-specific GM-BHQs.

DTI data were preprocessed using the FMRIB software library (FSL) 5.0.11^74^. First, we aligned all the diffuse images with the initial b0 image and used eddy correction to perform motion and eddy current distortion corrections. Following these modifications, the FA images were calculated using a DTIFit. FA images were then spatially normalized into the standard Montreal Neurological Laboratory (MNI) space using FLIRT and FNIRT. After spatial normalization, the data were smoothed with an 8 mm FWHM. Mean and SD images were generated from all FA images, and individual FA quotient images were calculated using the following formula: 100 + 15 × (individual FA-mean) / SD. Regional FA quotients were extracted using the Johns Hopkins University (JHU) DTI-based white matter atlas^75^ and averaged across regions to create a participant-specific FA-BHQ. See Nemoto et al. (2017)^21^ for more information. In our previous studies, whole-brain level GM-BHQ showed a positive correlation with dietary balance^23^,curiosity^24^, and behavioral activation^27^ as well as negatively correlated with fatigue^25^ and unhealthy lifestyles^26^. Similarly, it was shown that whole-brain FA-BHQ was positively correlated with cognitive function, fish diet intake^22^, housing quality, and anxiety^76^.

In addition to GM-BHQ and FA-BHQ, which are representative of the whole brain, we also defined 7 subregions of FABHQ from the perspective of the region of interest (ROI) since previous studies have elucidated the relationship between structural connectivity in these regions and emotional regulations^77–84^. Previous studies have shown that some regions of FA on the limbic-thalamo-cortical pathway including corona radiata, internal capsule, and cingulum are associated with emotional regulation^41–44^. On the other hand, others argue that the prefrontal cortex/uncinate fasciculus/amygdala pathway^85–87^ or the corpus callosum^88,89^ are more critical for emotional regulation. Therefore, correlation analysis with SHS and age is performed for these regions in the section below.

### Statistical analysis

Path analysis was performed to investigate the association between age, GM-BHQ, FA-BHQ, and SHS based on the hypothesis that GM-BHQ and FA-BHQ mediate the association between age and SHS. The level of statistical significance was set at *p* < 0.05. All statistical analyses were performed using SPSS/AMOS version 26 (IBM Corporation, Armonk, NY, USA).

## Results

**Figure 2:**
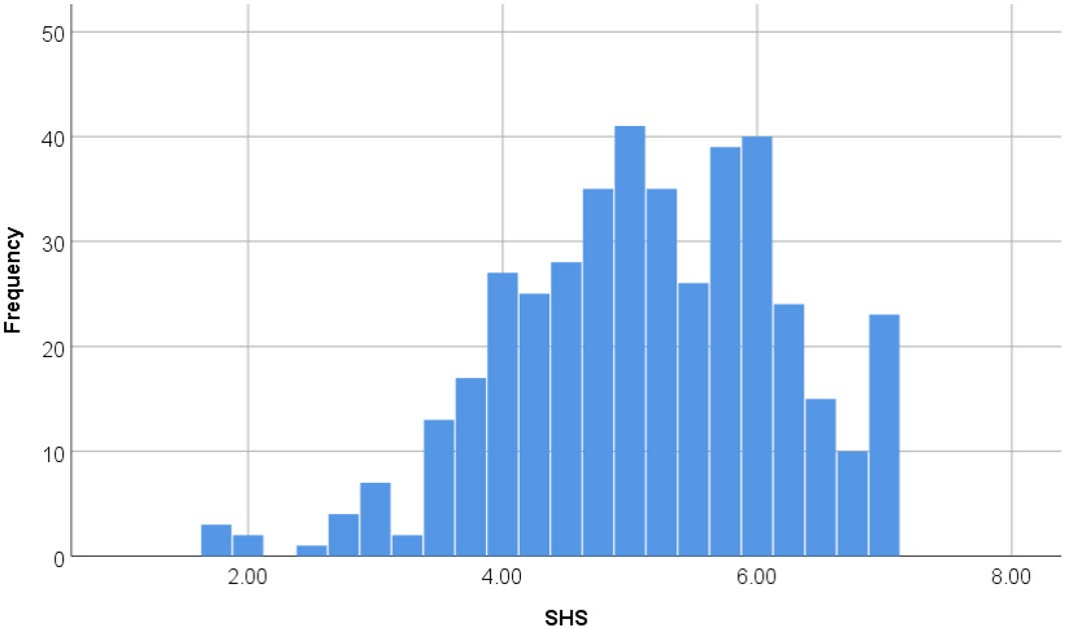
Distribution of SHS scores.

Figure 2 shows a histogram showing the distribution of SHS scores.

Table 1 indicates a statistically significant difference between institutes for SHS (t = 2.186, p < 0.05), age (t = 5.089, p < 0.001), income (t = 10.766, p < 0.001), FA-BHQ (t = 9.737, p < 0.001), sex (χ^2^ = 49.575, p < 0.001), and knowledge work (χ^2^ = 38.417, p < 0.001). Therefore, we decided to use the entire sample in a single model, controlling for institute in the following analyses.

**Table 1:** Statistical differences between institutes for participation.

	Kyoto		Tokyo		t	p
	Mean	SD	Mean	SD		
SHS	4.899	1.049	5.183	1.066	2.186	*
Age	46.830	7.123	41.890	10.411	5.089	***
BMI	22.563	3.375	23.277	3.689	1.685	
Income	6.3293	3.49622	10.9313	3.358	10.766	***
FA-BHQ	96.603	3.396	100.624	3.163	9.737	***
GM-BHQ	100.057	5.644	101.487	7.475	1.918	
	N	%	N	%	χ2	
Male	36	43.9%	274	81.8%	49.575	***
Female	46	56.1%	61	18.2%		
Knowledge work	20	24.4%	209	62.4%	38.417	***
Non-Knowledge work	62	75.6%	126	37.6%		

n = 417; * p < 0.05; ** p < 0.01; *** p < 0.001.

The descriptive statistics of all subjects and the correlation coefficients between the psychological scales are shown in Table 2. SHS was correlated with knowledge work (r = 0.124, p < 0.05), income (r = 0.241, p < 0.001), and the FA-BHQ scores (r = 0.134, p < 0.01). In contrast, age was correlated with income (r = 0.225, p < 0.001), FA-BHQ (r = − 0.386, p < 0.001), and GM-BHQ scores (r = − 0.627, p < 0.001). For FA-BHQ, a statistically significant correlation was shown at the 5% level with all variables except sex and BMI. For GM-BHQ, a statistically significant correlation was shown at the 5% level with all variables except SHS and knowledge work. In addition, as expected, a relatively high correlation was shown between knowledge work and income (r = 0.407, p < 0.001).

**Table 2:** Descriptive statistics and correlations.

	Variable	Mean	SD	1	2	3	4	5	6	7
1	SHS	5.127	1.068							
2	Age	42.860	10.039	− 0.008						
3	Sex	1.26	0.437	0.066	0.020					
4	BMI	23.137	3.635	− 0.031	0.059	− 0.355***				
5	Knowledge work	0.549	0.498	0.124*	0.036	− 0.119*	− 0.044			
6	Income	10.026	3.845	0.241***	0.225***	− 0.339***	0.088	0.407***		
7	FA-BHQ	99.833	3.583	0.134**	− 0.386***	− 0.057	− 0.052	0.195***	0.160**	
8	GM-BHQ	101.205	7.168	0.006	− 0.627***	0.264***	− 0.332***	0.003	− 0.204***	0.371***

n = 417; *p < 0.05; **p < 0.01; ***p < 0.001.

**Figure 3:**
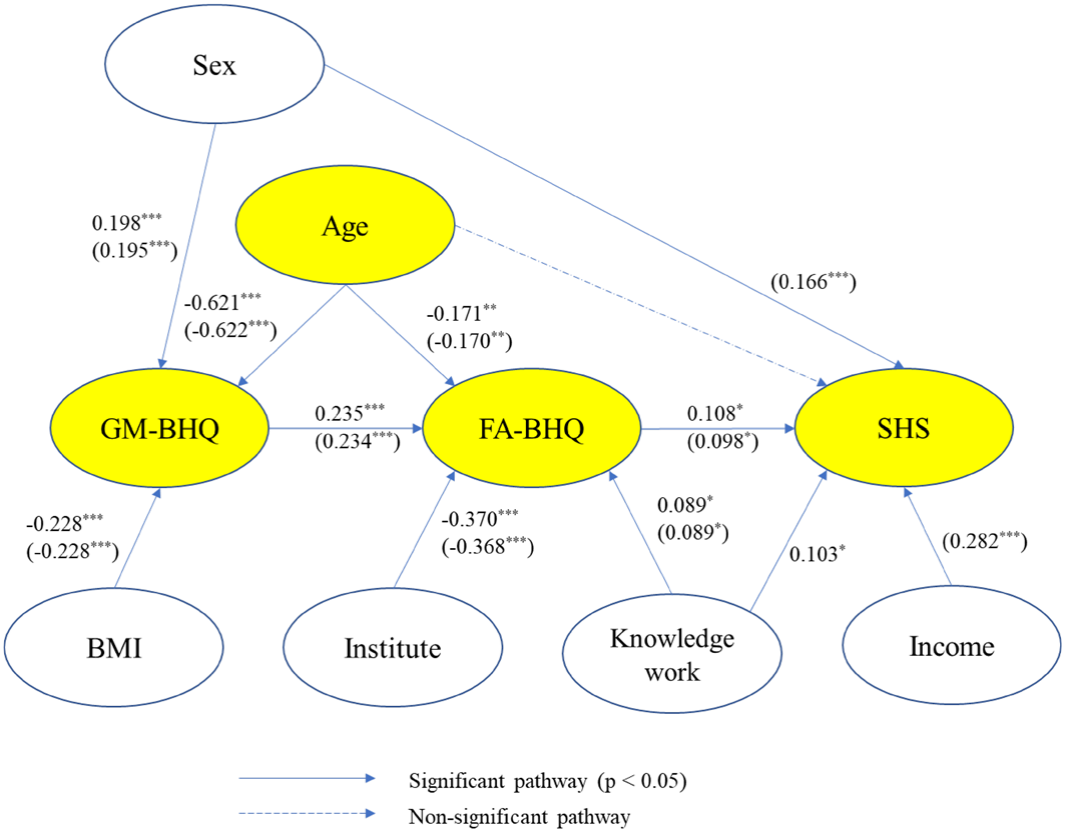
Path diagram for the resulting association between age, SHS, the GM-BHQ, and the FA-BHQ. Goodness-of-fit indices: χ2 = 9.606 (17.885); df = 12 (17); root mean square error of approximation (RMSEA) = 0.000 (0.011); probability of close fit (PCLOSE) = 0.982 (0.970); goodness of fit index (GFI) = 0.994 (0.991); adjusted goodness of fit index (AGFI) = 0.983 (0.975); normed fit index (NFI) = 0.986 (0.981); comparative fit index (CFI) = 1.000 (0.999). n = 417; *p < 0.05; **p < 0.01; ***p < 0.001. The figures are controlled for sex, BMI, knowledge work, and institute. The numbers in parentheses are the ones when income is added to the control. Error terms and correlations between variables are omitted in the figure. The yellow variables are the main variables.

To clarify the relationship among age, GM-BHQ, FA-BHQ, and SHS, we conducted path analysis as depicted in Fig. 3. Modification indices were used to improve the model fittings. For reference, the figures of the standardized path coefficient are also shown. The model’s goodness-of-fit indices (displayed in the figure legend) showed high adaptability. However, although FA-BHQ was directly associated with SHS, age and GM-BHQ were not; the latter had only an indirect association with SHS via FA-BHQ. Therefore, the total effect on SHS was 0.108 (0.098) for FA-BHQ, 0.025 (0.023) for GM-BHQ, and − 0.034 (− 0.031) for age, as calculated from the figures in Table 3 (The numbers in parentheses are the ones when income is used for control).

**Table 3:** Path coefficient.

Path			Path coefficient
GM-BHQ	⇒	FA-BHQ	0.235*** (0.234***)
Institute	⇒	FA-BHQ	− 0.370*** (− 0.368***)
Age	⇒	FA-BHQ	− 0.171** (− 0.170**)
Knowledge work	⇒	FA-BHQ	0.089* (0.089*)
Age	⇒	GM-BHQ	− 0.621*** (− 0.622***)
Sex	⇒	GM-BHQ	0.198*** (0.195***)
BMI	⇒	GM-BHQ	− 0.228*** (− 0.228***)
FA-BHQ	⇒	SHS	0.108* (0.098*)
Sex	⇒	SHS	nil (0.166***)
Knowledge work	⇒	SHS	0.103* (nil)
Income	⇒	SHS	nil (0.282***)
			Covariance
Age	⇔	Institute	0.199*** (0.193***)
Sex	⇔	Institute	0.348*** (0.316***)
BMI	⇔	Institute	− 0.104* (nil)
Income	⇔	Institute	nil (− 0.477***)
Income	⇔	Age	nil (0.224***)
BMI	⇔	Sex	− 0.358*** (− 0.321***)
Income	⇔	Sex	nil (− 0.323***)
Error of SHS	⇔	Sex	0.089* (nil)
Institute	⇔	Knowledge work	− 0.315*** (− 0.311***)
Sex	⇔	Knowledge work	− 0.134** (− 0.134**)
Income	⇔	Knowledge work	nil (0.399***)

n = 417; *p < 0.05; **p < 0.01; ***p < 0.001.

The figures are controlled for sex, BMI, knowledge work, and institute. The numbers in parentheses are the ones when income is added to the control.

Given that this study was conducted between multiple institutions, a multi-group analysis^90^ was performed to verify invariance in the magnitude of the path coefficients between the institutes. As a result, there were significant differences in the path from knowledge work to SHS in the model without income in control and from sex to SHS in the model with income in control between institutes at the 5% level. There were also significant differences between institutes at the 5% level in the correlation between sex and knowledge work in the model without income in control, sex and income in the model with income in control, and age and income in the model with income in control (details will be provided upon request). However, there was no significant difference of 5% level between institutes in the paths between the major variables, i.e., age, GM-BHQ, FA-BHQ, and SHS. This shows the robustness of the model shown in Fig. 3.

As shown in Table 4, FA-BHQ is still significantly correlated with SHS (r = 0.098, p < 0.05) and age (r = − 0.166, p < 0.01) at the 5% level even when controlled by sex, BMI, knowledge work, institute, GM-BHQ (and age for SHS). Moreover, the following 5 FA regions showed a significant correlation with SHS individually (p < 0.05, controlled for age, sex, BMI, knowledge work, institute, and GM-BHQ): the internal capsule (r = 0.115, p < 0.05), corona radiata (r = 0.127, p < 0.05), posterior thalamic radiation (r = 0.115, p < 0.05), cingulum (r = 0.103, p < 0.05), and superior longitudinal fasciculus (r = 0.118, p < 0.05). These variables were also statistically significant at the 5% level in the multiple comparisons using the Benjamini & Hochberg method. However, when income was added to the control, although each alone, except for Cingulum (r = 0.096, p < 0.10), still had a statistically significant correlation with SHS at the 5% level, none of them was statistically significant at the 5% level in the multiple comparisons using the Benjamini & Hochberg method. For GM-BHQ, no regions showed a significant correlation with SHS (p < 0.05, controlled for age, sex, BMI, knowledge work, and institute). This result was similar when income was put in the control.

**Table 4:** Partial correlations.

	Mean	SD	SHS	Age
FA-BHQ	99.833	3.583	0.098* (0.086)	− 0.166** (− 0.155**)
Corpus callosum	100.759	5.147	0.055 (0.046)	− 0.152**^☨^ (− 0.157**^☨^)
Internal capsule	100.344	4.979	0.115*^☨^ (0.107*)	− 0.189***^☨^ (− 0.194***^☨^)
Corona radiata	102.802	6.738	0.127*^☨^ (0.110*)	− 0.211***^☨^ (− 0.226***^☨^)
Posterior thalamic radiation	99.804	5.546	0.115*^☨^ (0.103*)	− 0.211***^☨^ (− 0.220***^☨^)
Cingulum	99.523	4.182	0.103*^☨^ (0.096)	− 0.105*^☨^ (− 0.112*^☨^)
Superior longitudinal fasciculus	100.231	4.506	0.118*^☨^ (0.105*)	− 0.175***^☨^ (− 0.187***^☨^)
Uncinate fasciculus	98.666	6.484	0.045 (0.036)	− 0.004 (− 0.015)

n = 417; *p < 0.05; **p < 0.01; ***p < 0.001.

^☨^p < 0.05 for multiple comparisons using the Benjamini and Hochberg method.

The figures are controlled for age, sex, BMI, knowledge work, institute, and GM-BHQ for SHS.

The figures are controlled for sex, BMI, knowledge work, institute, and GM-BHQ for age.

The numbers in parentheses are the ones when income is added to the control.

Contrastingly, 6 of 7 FA regions showed a significant correlation with age (p < 0.05, controlled for sex, BMI, knowledge work, institute, and GM-BHQ). Furthermore, all the GM regions correlated with age (p < 0.05, controlled for sex, BMI, knowledge work, and institute). These results did not change much with the multiple comparisons by the Benjamini & Hochberg method, and even with income in the control.

**Figure 4:**
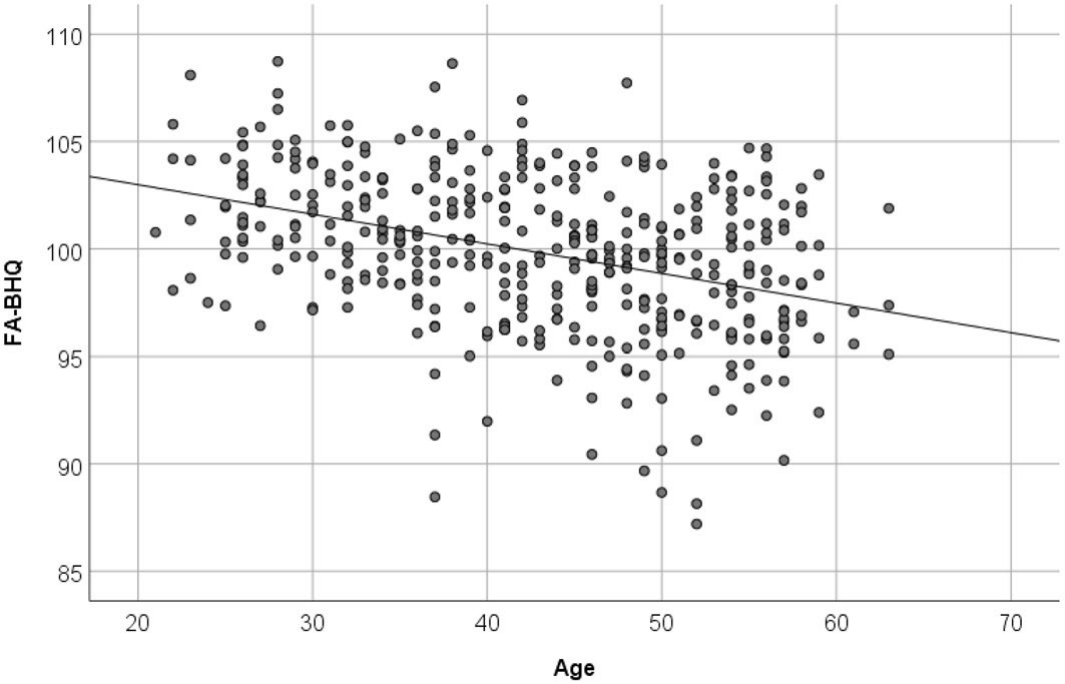
Scatter plot showing the relationship between age and FA-BHQ.

**Figure 5:**
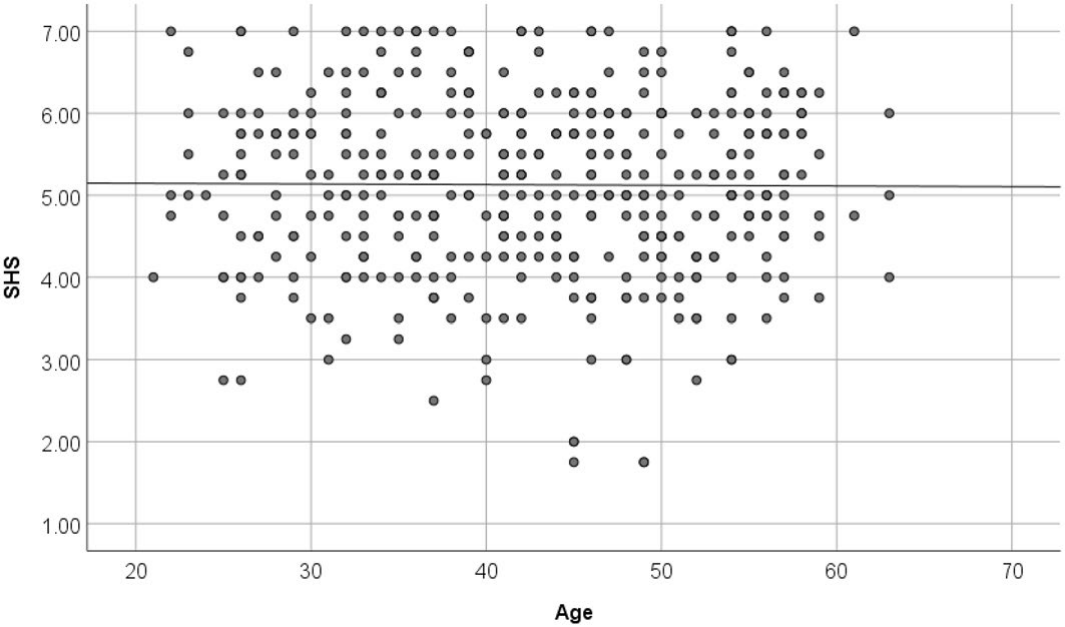
Scatter plot showing the relationship between age and SHS.

**Figure 6:**
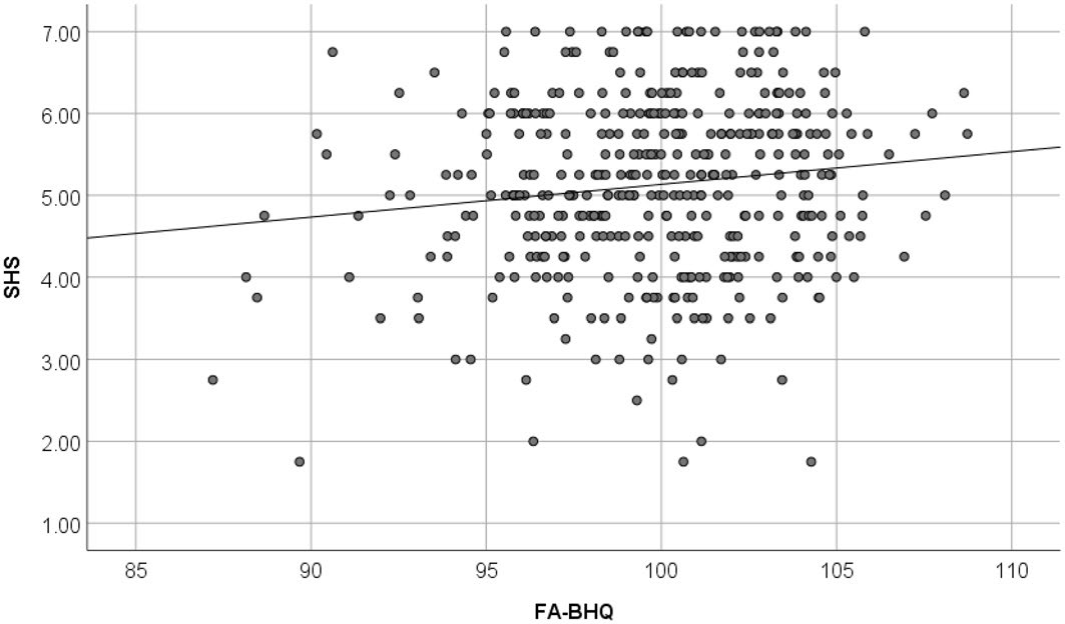
Scatter plot showing the relationship between FA-BHQ and SHS.

**Figure 7:**
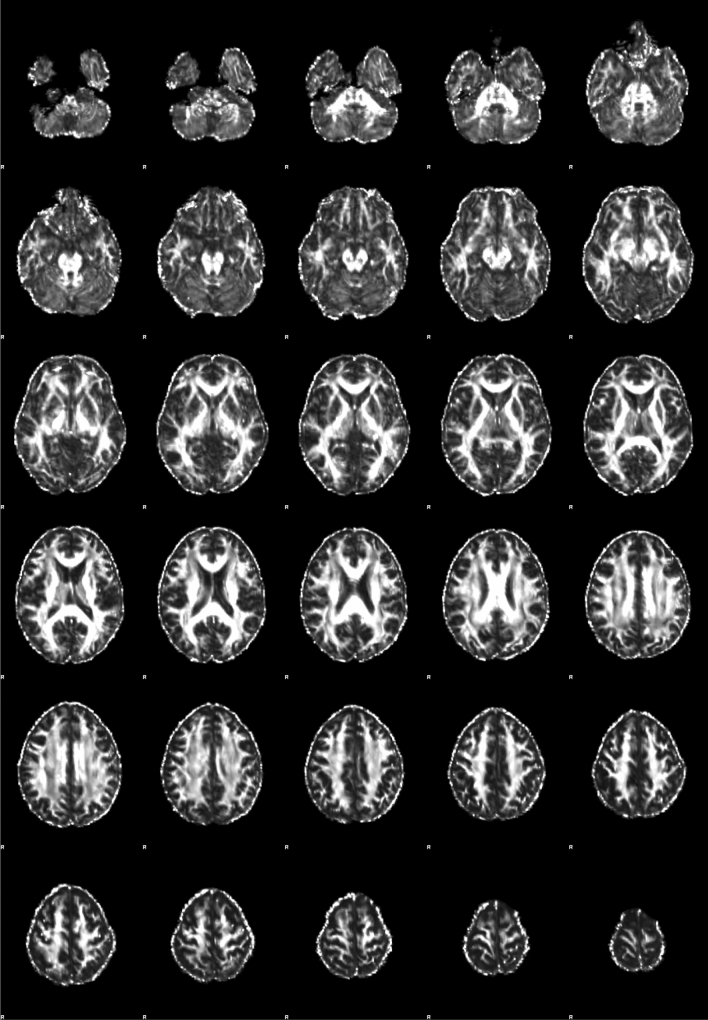
MRI slices of subjects with the smallest FA-BHQ.

**Figure 8:**
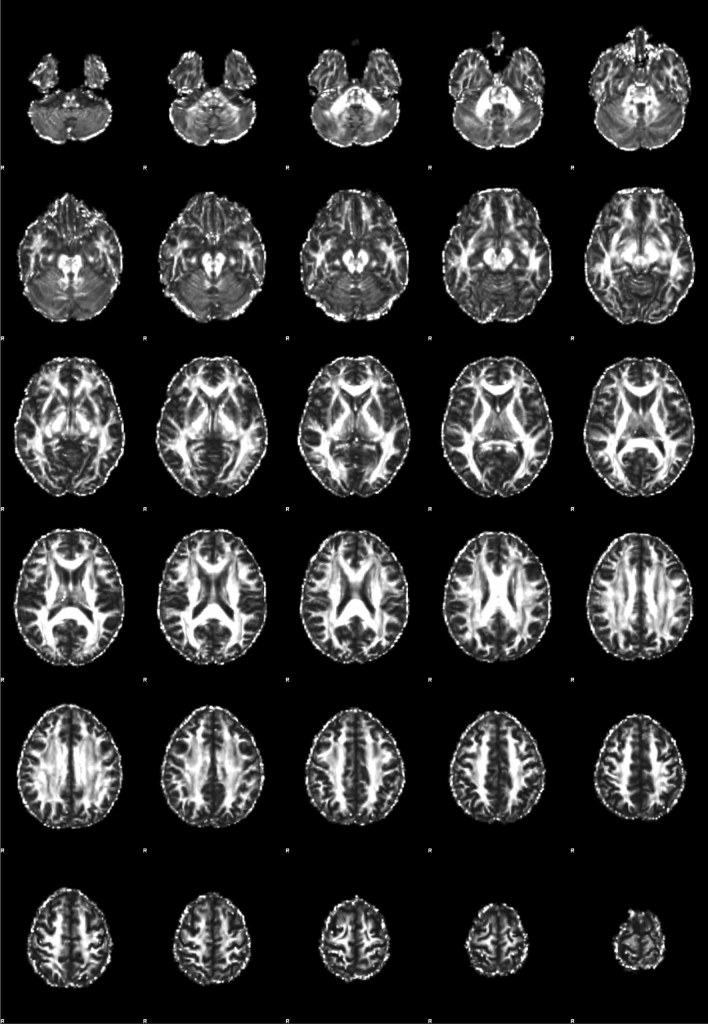
MRI slices of subjects with the largest FA-BHQ.

Figures 4, 5, and 6 are a scatter plot showing the relationship between age and FA-BHQ, age and SHS, and FA-BHQ and SHS, respectively. The largest correlation is the relationship between age and FA-BHQ (Fig. 4). In addition, although it is smaller than this, a correlation can be visually confirmed between FA-BHQ and SHS (Fig. 6). However, there is almost no correlation between age and SHS, so the regression line is almost flat (Fig. 5). Figures 7 and 8 show MRI slices for subjects with the smallest and largest FA-BHQ, respectively.

## Discussion

As the population ages, the realization of a society where people can live happily while getting older has become an urgent issue in many countries. However, many previous studies using SHS, a representative scale of happiness, showed no significant correlation between happiness and age^2,7–12,17^. On the other hand, large studies using scales related to SHS have shown that age correlates with life satisfaction and well-being in a U-shape^13–16^. In any case, it can be said that happiness and well-being do not tend to decrease consistently with age, which is almost common in many studies. This is seemingly inconsistent with studies showing that the brain atrophies with age^21–26^, and studies showing that the brain correlates with happiness^27–31^.

The current study showed that brain structures, that is, GM-BHQ and FA-BHQ, had a significant negative correlation with age. At the same time, most regions tested had a negative correlation with age. However, the correlation between FA-BHQ and age was smaller than the correlation between GM-BHQ and age. On the other hand, SHS showed a significant positive correlation with FA-BHQ and some of its subscales. Additionally, as reported in previous studies, SHS did not show a direct correlation with age. This may answer some unexplained parts of previous studies that showed the brain declines with age, but the sense of happiness, which correlates with the brain, does not. That is, in the brain, both GM-BHQ and FA-BHQ tended to decline widely with aging, with the former stronger than the latter, whereas only FA-BHQ correlated with happiness. As a result, it is thought that the effect of aging on happiness is considerably diminished. In an analysis we conducted in the past, which is consistent with this study, FA was shown to be correlated with measures close to happiness, such as subjective well-being and post-materialism (a personality that prefers mental affluence to physical affluence), but GM showed no correlation with these^21^. In the same vein, recent studies have also shown that measures such as well-being and life satisfaction, which are close to happiness, correlate with indicators related to white matter including FA^63,91^.

GM volume reduction is thought to be associated with neuronal aging. That is, a decrease in neurogenesis in the aged brain leads to a decrease in tissue integrity, function, and maintenance of the regenerative response. At the same time, the reduction of metabolism of neurons in the cerebral cortex, cerebellar cortex, hippocampus, basal ganglia, locus coeruleus, and substantia nigra leads to a decline in the number of synapses, which is thought to disturb plasticity and lead to brain atrophy^92^. On the other hand, the loss of white matter integrity in aging is explained primarily by atrophy and lesion formation, not by the aging process itself^93^. This difference between the two is a possible reason why GM-BHQ has a higher correlation with age than FA-BHQ.

This study also showed that the FA values of the internal capsule, corona radiata, posterior thalamic radiation, cingulum, and superior longitudinal fasciculus were significantly correlated with SHS controlled for sex, age, BMI, knowledge work, institute, and GM-BHQ. This result did not change much with multiple comparisons or with income control, although it was not significant when multiple comparisons and income control were performed at the same time. Here, the mechanism occurring in the brain is considered as follows: The cingulate sends projections to the amygdala and prefrontal cortex via the cingulum bundle, which extends from the prefrontal cortex to the entire medial collateral limbic complex including parahippocampally^77^, and plays a central role in emotional processing such as evaluation, cognitive reassessment, emotional expression, and emotional response generation^78,79^.

Superior longitudinal fasciculus is an important bundle of associated fibers in the white matter of each cerebral hemisphere connecting the parietal, occipital, and temporal lobes with the ipsilateral frontal lobe^82^. Therefore, superior longitudinal fasciculus is considered to facilitate the formation of a bidirectional neural network that is necessary for core processes, such as attention, memory, emotions, and language^83,84^. Contrastingly, corona radiata is part of the limbic-thalamo-cortical circuits and includes thalamic projections from the internal capsule, the pathway to the thalamus and the amygdala^80,81^, to the cortex, which includes the prefrontal cortex GM areas that have been associated with top-down emotion regulation systems^94^. Therefore, corona radiata has been implicated in emotional regulation^43^.

In this way, the relationship between FA and happiness shown in this study can be said to be consistent considering the role that each region is thought to play. Previous studies have focused on and studied GM volume as a factor that influences happiness and well-being. However, consistent results have not been obtained between studies. It has been said that this is due to a difference in culture and scale used^40^. However, as different GM regions also correlated with SHS in studies of Japanese^28,30^, the need to test for factors other than culture and scale increased. In this study, with more study participants than previous studies, it was shown that FA, which is a measure of the integrity of white matter connecting each brain region, is a factor that influences SHS. This result presents a new research theme to be tackled in future research on the brain and happiness/well-being. In other words, when focusing on GM, consistent results may not be obtained due to the nature of the collected subjects, but by focusing on the communication path connecting GM, a consistent tendency may be found in the relationship between the brain and SHS.

Although neuroscience research in recent years has not elucidated the mechanism, FA in the region related to cognition and emotional regulation shown above has also been related to strong stress, such as PTSD and MDD^43,95–101^. This suggests that people with higher FA values ​​in the regions involved in cognitive and emotional regulation are less likely to experience strong stress and, as are shown in the current research, more likely to experience higher happiness. This is strangely consistent with the results of previous psychological studies, which showed that people with a higher ability to read and evaluate the emotions of others had lower stress^102–104^ and higher SHS^105,106^, and that people with lower stress tend to have higher SHS^107–109^. Previous studies have hypothesized that aging does not reduce happiness because gaining life experience increases certain tolerances^17,18^. Therefore, the mechanism behind it can be inferred from the results of this study: people who improve their ability to read the emotions of others as they get older maintain a high FA value for their age, so they are less likely to be stressed and feel happy.

This is the first study to show that FA levels mediate the relationship between aging and happiness. However, this study shows consistent results with the relationship between FA and emotional regulation shown by some previous neuroscience studies, as well as the relationship between emotional regulation and happiness shown by some psychological studies. Therefore, this research contributes to bridging these previous studies. The results of this study indicate that neuroscience may contribute to the policy issue of enhancing the happiness of a wide range of age groups, including the elderly. For reference, in our previous paper, the longitudinal analysis showed that certain types of training can prevent whole-brain FA reduction and burnout^110^. By developing and implementing such efforts, it may be possible to maintain the health of people's brains and enhance their happiness and well-being.

## Limitation

The current study has roughly five limitations. First, in this study, the age range was from 21 to 63 years, and it did not include many elderly people. However, studies involving more older people showed a statistically significant negative correlation between age and FA, while a statistically significant positive correlation between well-being and FA^21,63,91^. In addition, many studies, including the elderly, have shown that there is no statistically significant correlation between age and SHS^2,7–11^. From these, it is expected that the results shown by this study that FA regulates SHS more directly than age will be reproduced even if a study involving more elderly people is conducted. In any case, it is hoped that future studies will verify the reproducibility of the results of this study using samples that include more elderly people.

Next is the number and type of control variables. In this study, BMI, knonwledge work, and income were added to the control variables, but other variables (e.g., marital status, stress, and overall health) were not because they were not used in previous studies of the relation between brain and well-being although they were often used in studies of well-being^111–113^. In addition, this study mainly targeted employees. Therefore, in future studies, these variables should be added to the control variables for analysis using a wider range of samples to verify the reproducibility of the results of this study.

Third, it should also be noted that the results of this study were not all consistent with expectations. That is, no correlation was found with SHS for the corpus callosum that connects the left and right cerebral hemispheres, and the uncinate fasciculus that connects the gyrus of the frontal lobe to the anterior end of the temporal lobe. This result contradicts, for example, in the light of studies in which uncinate fasciculus is a critical pathway for anxiety^114^. However, it is consistent with Sanjuan and colleagues’ work showing that corona radiata, internal capsule, and cingulum play a greater role in PTSD than uncinate fasciculus in the pathway connecting the prefrontal cortex, thalamus, and amygdala^43^. In any case, the relationship between well-being and individual brain regions should be verified by further research in the future.

Fourth is about the definition of the mediation effect. In this study, FA-BHQ completely mediated the relationship between age and SHS, which is called the mediation effect. However, there is a view that mediation does not occur when there is no correlation between the starting point (age) and the ending point (SHS) as in this study^115^. Since there are pros and cons to this view, the results of this study should be verified by researchers in various positions.

The last is about the number of samples, research subjects, and analysis methods. The number of participants exceeding 400 in this study is higher than that in the related previous studies, but it is not high enough to guarantee generalizability. Moreover, this study is intended only for Japanese people. Besides, this study is a cross-sectional analysis and does not show a causal relationship. Therefore, future studies should verify the results of this study by targeting other ethnic groups with more samples and by using other analytical methods such as longitudinal analysis.

## Conclusion

In the current study, FA correlated with happiness, especially in the areas involved in emotional regulation, such as internal capsule, corona radiata, posterior thalamic radiation, cingulum, and superior longitudinal fasciculus. Contrastingly, age correlated with many GM and FA regions, but not with happiness. This means that brain conditions mediate the association between aging and happiness. Unlike previous brain studies that focused on GM, this study showed that the quality of the pathway connecting brain regions measured by FA is a factor that directly affects happiness.

## Data Availability

The datasets generated during the current study are available from the corresponding author on reasonable request.

## References

[1] Lansford, J.E. 2018. A lifespan perspective on subjective wellbeing, in *Handbook of Well-Being* (eds. Diener, E. Oishi, S. & Tay, L.) (Salt Lake City, UT: DEF Publishers, 2018). https://www.nobascholar.com/chapters/25/download.pdf

[2] Lyubomirsky S, Lepper HS (1999). A measure of subjective happiness: Preliminary reliability and construct validation. Soc. Indic. Res..

[3] Iani L, Lauriola M, Layous K, Sirigatti S (2014). Happiness in Italy: translation, factorial structure and norming of the subjective happiness scale in a large community sample. Soc. Indic. Res..

[4] Extremera N, Fernández-Berrocal P (2014). The Subjective Happiness Scale: Translation and preliminary psychometric evaluation of a Spanish version. Soc. Indic. Res..

[5] Simons M, Peeters S, Janssens M, Lataster J, Jacobs N (2018). Does age make a difference? Age as moderator in the association between time perspective and happiness. J. Happiness Stud..

[6] Vera-Villarroel P, Celis-Atenas K, Córdova-Rubio N (2011). Evaluation of happiness: psychometric analysis of the Subjective Happiness Scale in Chilean population. Terapia Psicol..

[7] Doğan T, Totan T (2013). Psychometric properties of Turkish version of the Subjective Happiness Scale. J. Happiness Well-being..

[8] Moghnie L, Kazarian SS (2012). Subjective happiness of Lebanese college youth in Lebanon: factorial structure and invariance of the Arabic subjective happiness scale. Soc. Indic. Res..

[9] Nan H (2014). Psychometric evaluation of the Chinese version of the subjective happiness scale: evidence from the Hong Kong FAMILY Cohort. Int. J. Behav. Med..

[10] Spagnoli P, Caetano A, Silva A (2012). Psychometric properties of a Portuguese version of the Subjective Happiness Scale. Soc. Indic. Res..

[11] Swami V (2008). Translation and validation of the Malay Subjective Happiness Scale. Soc. Indic. Res..

[12] Zager Kocjan, G., Jose, P. E., Sočan, G. & Avsec, A. Measurement invariance of the Subjective Happiness Scale across countries, gender, age, and time. *Assessment*. A-head-of-print (2021). 10.1177/1073191121993558 PMC9047108 33576241

[13] Blanchflower, D.G. & Graham, C.L. The mid-life dip in well-being: a critique. *Soc. Indic. Res*. A-head-of-print (2021). 10.1007/s11205-021-02773-w PMC8525618 34690403

[14] Blanchflower DG, Graham CL (2021). The U shape of happiness: a response. Perspect. Psychol. Sci..

[15] Easterlin RA (2003). Explaining happiness. Proc. Natl. Acad. Sci..

[16] Frijters P, Beatton T (2012). The mystery of the U-shaped relationship between happiness and age. J. Econ. Behav. Organ..

[17] McMahon E, Estes D (2012). Age-related differences in lay conceptions of well-being and experienced wellbeing. J. Happiness Stud..

[18] Carstensen LL, DeLiema M (2018). The positivity effect: a negativity bias in youth fades with age. Curr. Opin. Behav. Sci..

[19] Chien CL (2020). The Chinese version of the Subjective Happiness Scale: validation and convergence with multidimensional measures. J. Psychoeduc. Assess..

[20] Swami V (2009). Psychometric evaluation of the Tagalog and German Subjective Happiness Scales and a cross-cultural comparison. Soc. Indic. Res..

[21] Nemoto K, Oka H, Fukuda H, Yamakawa Y (2017). MRI-based Brain Healthcare Quotients: a bridge between neural and behavioral analyses for keeping the brain healthy. PLoS ONE.

[22] Kokubun K, Nemoto K, Yamakawa Y (2020). Fish intake may affect brain structure and improve cognitive ability in healthy people. Front. Aging Neurosci..

[23] Kokubun K, Yamakawa Y (2019). Association between food patterns and gray matter volume. Front. Hum. Neurosci..

[24] Kokubun K, Yamakawa Y, Hiraki K (2020). Association between behavioral ambidexterity and brain health. Brain. Sci..

[25] Kokubun K (2018). Association of fatigue and stress with gray matter volume. Front. Behav. Neurosci..

[26] Kokubun K, Pineda JCD, Yamakawa Y (2021). Unhealthy lifestyles and brain condition: Examining the relations of BMI, living alone, alcohol intake, short sleep, smoking, and lack of exercise with gray matter volume. PLoS ONE.

[27] Kokubun, K., Yamakawa, Y. & Nemoto, K. The link between the brain volume derived index and the determinants of social performance. *Curr. Psychol*. a-head-of-print (2022).

[28] Matsunaga M (2016). Structural and functional associations of the rostral anterior cingulate cortex with subjective happiness. Neuroimage.

[29] Lewis GJ, Kanai R, Rees G, Bates TC (2014). Neural correlates of the ‘good life’: Eudaimonic well-being is associated with insular cortex volume. Soc. Cogn. Affect. Neurosci..

[30] Sato W (2015). The structural neural substrate of subjective happiness. Sci. Rep..

[31] Yang J, Wei D, Wang K, Qiu J (2013). Gray matter correlates of dispositional optimism: a voxel-based morphometry study. Neurosci. Lett..

[32] Kong F (2015). Examining gray matter structures associated with individual differences in global life satisfaction in a large sample of young adults. Soc. Cogn. Affect. Neurosci..

[33] Kubarych TS (2012). A multivariate twin study of hippocampal volume, self-esteem and well-being in middle-aged men. Genes Brain Behav..

[34] Pruessner JC (2005). Self-esteem, locus of control, hippocampal volume, and cortisol regulation in young and old adulthood. Neuroimage.

[35] Damasio AR (2000). Subcortical and cortical brain activity during the feeling of self-generated emotions. Nat. Neurosci..

[36] George MS, Ketter TA, Parekh PI, Herscovitch P, Post RM (1996). Gender differences in regional cerebral blood flow during transient self-induced sadness or happiness. Biol. Psych..

[37] Habel U, Klein M, Kellermann T, Shah NJ, Schneider F (2005). Same or different? Neural correlates of happy and sad mood in healthy males. Neuroimage.

[38] Mitterschiffthaler MT, Fu CH, Dalton JA, Andrew CM, Williams SC (2007). A functional MRI study of happy and sad affective states induced by classical music. Hum. Brain Mapp..

[39] Schwartz GE, Davidson RJ (1997). Neuroanatomical correlates of happiness, sadness, and disgust. Am. J. Psych..

[40] Machado L, Cantilino A (2017). Neural correlates of wellbeing scales preliminary data. Aust. N. Z. J. Psych..

[41] Lanius RA (2001). Neural correlates of traumatic memories in posttraumatic stress disorder: a functional MRI investigation. Am. J. Psych..

[42] Lanius RA (2003). Recall of emotional states in posttraumatic stress disorder: an fMRI investigation. Biol. Psych..

[43] Sanjuan PM, Thoma R, Claus ED, Mays N, Caprihan A (2013). Reduced white matter integrity in the cingulum and anterior corona radiata in posttraumatic stress disorder in male combat veterans: a diffusion tensor imaging study. Psych. Res..

[44] Schuff N (2011). Patterns of altered cortical perfusion and diminished subcortical integrity in posttraumatic stress disorder: an MRI study. Neuroimage.

[45] Satici, S. A., Kayis, A. R., Satici, B., Griffiths, M. D. & Can, G. Resilience, Hope, and Subjective Happiness Among the Turkish Population: Fear of COVID-19 as a Mediator. *Int. J. Ment. Health Addict*. a-head-of-print, 1–16 (2020). 10.1007/s11469-020-00443-5 PMC7714252 33293904

[46] Morales-Vives F, Dueñas JM, Vigil-Colet A, Camarero-Figuerola M (2020). Psychological variables related to adaptation to the COVID-19 lockdown in Spain. Front. Psychol..

[47] Kawamichi H (2016). Being in a romantic relationship is associated with reduced gray matter density in striatum and increased subjective happiness. Front. Psychol..

[48] Sato W (2019). Resting-state neural activity and connectivity associated with subjective happiness. Sci. Rep..

[49] Katsumi Y, Kondo N, Dolcos S, Dolcos F, Tsukiura T (2021). Intrinsic functional network contributions to the relationship between trait empathy and subjective happiness. Neuroimage.

[50] Babu MG, Kadavigere R, Koteshwara P, Sathian B, Rai KS (2020). Rajyoga meditation induces grey matter volume changes in regions that process reward and happiness. Sci. Rep..

[51] Kong F (2019). Neural correlates of social well-being: Gray matter density in the orbitofrontal cortex predicts social well-being in emerging adulthood. Soc. Cogn. Affect. Neurosci..

[52] Li R, Zhu X, Zheng Z, Wang P, Li J (2020). Subjective well-being is associated with the functional connectivity network of the dorsal anterior insula. Neuropsychologia.

[53] Luo Y (2014). Regional homogeneity of intrinsic brain activity in happy and unhappy individuals. PLoS ONE.

[54] Cantril, H. The patterns of human concerns (Rutgers University Press, 1965).

[55] Buhrmann A (2021). Cerebellar grey matter volume in older persons is associated with worse cognitive functioning. Cerebellum.

[56] Diener, E., Kahneman, D., Tov, W., & Arora, R. Income’s differential impact on judgments of life versus affective well-being in International differences in *Well-being* (eds. Diener, E., Kahneman, D., & Helliwell, J.) 3–15. (Oxford University Press, 2010).

[57] Oishi S, Schimmack U (2010). Culture and well-being: a new inquiry into the psychological wealth of nations. Perspect. Psychol. Sci..

[58] Stevenson, B., & Wolfers, J. Economic growth and subjective well-being: Reassessing the Easterlin Paradox (Brookings Papers on Economic Activity, 2008).

[59] Chen HL (2020). The association between physical fitness performance and subjective happiness among Taiwanese adults. Int. J. Environ. Res. Public Health..

[60] Kawashima M (2015). Associations between subjective happiness and dry eye disease: a new perspective from the Osaka study. PLoS ONE.

[61] Matsunaga M, Isowa T, Yamakawa K, Ohira H (2013). Association between the serotonin transporter polymorphism (5HTTLPR) and subjective happiness level in Japanese adults. Psychol. Well-Being: Theory Res. Pract..

[62] Gutin I (2018). In BMI we trust: reframing the body mass index as a measure of health. Soc. Theory Health..

[63] Maeda CT (2021). Brain microstructural properties related to subjective well-being: diffusion tensor imaging analysis. Soc. Cogn. Affect. Neurosci..

[64] Hauser RM (2002). Meritocracy, cognitive ability, and the sources of occupational success.

[65] Alshehri AA (2016). Subjective happiness assessment among Taif University medical students. Am. J. Edu. Res..

[66] Sun S, Chen J, Johannesson M, Kind P, Burström K (2016). Subjective well-being and its association with subjective health status, age, sex, region, and socio-economic characteristics in a Chinese population study. J. Happiness Stud..

[67] Noble KG (2015). Family income, parental education and brain structure in children and adolescents. Nat. Neurosci..

[68] Tomasi D, Volkow ND (2021). Associations of family income with cognition and brain structure in USA children: prevention implications. Mol. Psych..

[69] Diener E, Seligman ME (2004). Beyond money: toward an economy of well-being. Psychol. Sci. Public Interest.

[70] Nan H (2014). Psychometric evaluation of the Chinese version of the Subjective Happiness Scale: evidence from the Hong Kong FAMILY Cohort. Int. J. Behav. Med..

[71] Ballonoff Suleiman A, Johnson M, Shirtcliff EA, Galván A (2015). School-based sex education and neuroscience: what we know about sex, romance, marriage, and adolescent brain development. J. Sch. Health..

[72] Ashburner J (2007). A fast diffeomorphic image registration algorithm. Neuroimage.

[73] Tzourio-Mazoyer N (2002). Automated anatomical labeling of activations in SPM using a macroscopic anatomical parcellation of the MNI MRI single-subject brain. Neuroimage.

[74] Jenkinson, M., Beckmann, C. F., Behrens, T. E., Woolrich, M. W. & Smith, S. M. *Fsl. Neuroimage* **62**(2), 782-790 (2012). 10.1016/j.neuroimage.2011.09.015 21979382

[75] Mori S (2008). Stereotaxic white matter atlas based on diffusion tensor imaging in an ICBM template. Neuroimage.

[76] Pineda JCD, Kokubun K, Ikaga T, Yamakawa Y (2021). Housing quality and behavior affect brain health and anxiety in healthy Japanese adults. Sci. Rep..

[77] Pandya DN, Van Hoesen GW, Mesulam MM (1981). Efferent connections of the cingulate gyrus in the rhesus monkey. Exp. Brain Res..

[78] Etkin A, Egner T, Kalisch R (2011). Emotional processing in anterior cingulate and medial prefrontal cortex. Trends Cogn. Sci..

[79] Ochsner KN (2009). Bottom-up and top-down processes in emotion generation: common and distinct neural mechanisms. Psychol. Sci..

[80] Makris, N. *et al*. MRI-based topographic parcellation of human cerebral white matter and nuclei: II. Rationale and applications with systematics of cerebral connectivity. *Neuroimage*. **9**, 18–45 (1999). 10.1006/nimg.1998.0384 9918726

[81] Zikopoulos B, Barbas H (2012). Pathways for emotions and attention converge on the thalamic reticular nucleus in primates. J. Neurosci..

[82] Schmahmann, J. D., Smith, E. E., Eichler, F. S. & Filley, C. M. Cerebral white matter: neuroanatomy, clinical neurology, and neurobehavioral correlates. *Ann. N.Y. Acad. Sci*. **1142**, 266 (2008). 10.1196/annals.1444.017 PMC3753195 18990132

[83] Mesulam MM (1998). From sensation to cognition. Brain.

[84] Petrides M, Pandya DN (2002). Comparative cytoarchitectonic analysis of the human and the macaque ventrolateral prefrontal cortex and corticocortical connection patterns in the monkey. Eur. J. Neurosci..

[85] Ayling E, Aghajani M, Fouche JP, van der Wee N (2012). Diffusion tensor imaging in anxiety disorders. Curr. Psych. Rep..

[86] Kim MJ, Whalen PJ (2009). The structural integrity of an amygdala–prefrontal pathway predicts trait anxiety. J. Neurosci..

[87] Von Der Heide RJ, Skipper LM, Klobusicky E, Olson IR (2013). Dissecting the uncinate fasciculus: disorders, controversies and a hypothesis. Brain.

[88] Konrad A (2012). Broad disruption of brain white matter microstructure and relationship with neuropsychological performance in male patients with severe alcohol dependence. Alcohol Alcohol..

[89] Müller-Oehring EM, Schulte T, Fama R, Pfefferbaum A, Sullivan EV (2009). Global–local interference is related to callosal compromise in alcoholism: a behavior-DTI association study. Alcohol. Clin. Exp. Res..

[90] Byrne BM (2004). Testing for multigroup Invariance using AMOS graphics: a road less traveled. Struct. Equ. Model..

[91] Cabeen, R.P., Toga, A.W. & Allman, J.M. Frontoinsular cortical microstructure is linked to life satisfaction in young adulthood. *Brain Imaging Behav*. A-head-of-print (2021). 10.1007/s11682-021-00467-y PMC9229410 33825124

[92] Dorszewska J (2013). Cell biology of normal brain aging: synaptic plasticity–cell death. Aging Clin. Exp. Res..

[93] Vernooij MW (2008). White matter atrophy and lesion formation explain the loss of structural integrity of white matter in aging. Neuroimage.

[94] Karababa IF, Bayazıt H, Kılıçaslan N, Celik M, Cece H, Karakas E, Selek S (2015). Microstructural changes of anterior corona radiata in bipolar depression. Psych. Investig..

[95] van Velzen LS (2020). White matter disturbances in major depressive disorder: a coordinated analysis across 20 international cohorts in the ENIGMA MDD working group. Mol. Psych..

[96] Wu F (2011). Whiter matter abnormalities in medication-naive subjects with a single short-duration episode of major depressive disorder. Psych. Res. Neuroimag..

[97] O'Doherty DC (2018). White matter integrity alterations in post-traumatic stress disorder. Hum. Brain Mapp..

[98] Choi S (2015). Association of brain-derived neurotrophic factor DNA methylation and reduced white matter integrity in the anterior corona radiata in major depression. J. Affect. Disord..

[99] Cui Y (2020). White matter microstructural differences across major depressive disorder, bipolar disorder and schizophrenia: A tract-based spatial statistics study. J. Affect. Disord..

[100] Zhang A (2013). White matter tract integrity of anterior limb of internal capsule in major depression and type 2 diabetes. Neuropsychopharmacology.

[101] Liao Y (2013). Is depression a disconnection syndrome? Meta-analysis of diffusion tensor imaging studies in patients with MDD. J. Psych. Neurosci..

[102] Martínez-Monteagudo MC, Inglés CJ, Granados L, Aparisi D, García-Fernández JM (2019). Trait emotional intelligence profiles, burnout, anxiety, depression, and stress in secondary education teachers. Pers. Individ. Differ..

[103] Partido BB, Owen J (2020). Relationship between emotional intelligence, stress, and burnout among dental hygiene students. J. Dent. Educ..

[104] Ruiz-Aranda D, Extremera N, Pineda-Galan C (2014). Emotional intelligence, life satisfaction and subjective happiness in female student health professionals: the mediating effect of perceived stress. J. Psychiatr. Ment. Health Nurs..

[105] Kugbey N, Atefoe EA, Anakwah N, Nyarko K, Atindanbila S (2018). Emotional intelligence and personal growth initiative effects on subjective happiness among university students. J. Psychol. Afr..

[106] Szczygieł D, Mikolajczak M (2017). Why are people high in emotional intelligence happier? They make the most of their positive emotions. Pers. Individ. Differ..

[107] González-Ramírez MT, Landero-Hernández R, Quezada-Berumen L, Ibarra-González L (2017). Stressful situations affecting the perception of happiness: Love as a stressor. Ansiedad y Estrés..

[108] Schiffrin HH, Nelson SK (2010). Stressed and happy? Investigating the relationship between happiness and perceived stress. J. Happiness Stud..

[109] Silva RG, Figueiredo-Braga M (2018). Evaluation of the relationships among happiness, stress, anxiety, and depression in pharmacy students. Curr. Pharm. Teach. Learn..

[110] Kokubun K, Ogata Y, Koike Y, Yamakawa Y (2020). Brain condition may mediate the association between training and work engagement. Sci. Rep..

[111] Carr D, Freedman VA, Cornman JC, Schwarz N (2014). Happy marriage, happy life? Marital quality and subjective well-being in later life. J. Marriage Fam..

[112] Zhu X (2018). Together means more happiness relationship status moderates the association between brain structure and life satisfaction. Neuroscience.

[113] Steptoe A, Deaton A, Stone AA (2015). Subjective wellbeing, health, and ageing. Lancet.

[114] Kim MJ, Whalen PJ (2009). The structural integrity of an amygdala–prefrontal pathway predicts trait anxiety. J. Neurosci..

[115] Baron RM, Kenny DA (1986). The moderator–mediator variable distinction in social psychological research: Conceptual, strategic, and statistical considerations. J. Pers. Soc. Psychol..

